# Rapid intravenous opioid titration for acute symptom crises in palliative care: a real-world retrospective study

**DOI:** 10.1007/s00520-026-11245-1

**Published:** 2026-09-23

**Authors:** Kevin Nasrinfar, Valentine Simonet, Andrea Trippini, Tito Bosia, Eduardo Bruera, Claudia Gamondi

**Affiliations:** 1 Service Des Soins Palliatifs Et de Support, CHUV Centre Hospitalier Universitaire Vaudois, Av. de Beaumont 29, 1012 Lausanne, Switzerland; 2 Department of Palliative, Rehabilitation and Integrative Medicine, The University of Texas MD Anderson Cancer Center, Houston, TX USA

**Keywords:** Palliative care, Opioids, Intravenous titration

## Abstract

**Context:**

Rapid intravenous opioid titration is commonly used to manage acute symptom crises in palliative care, yet data describing its implementation and outcomes in routine practice remain limited.

**Objectives:**

To evaluate short-term symptom outcomes, immediate tolerability, and patient-reported satisfaction associated with rapid intravenous opioid titration in palliative care patients experiencing acute pain or dyspnea crises.

**Methods:**

We conducted a retrospective single-center study including 38 hospitalized palliative care patients who underwent rapid intravenous opioid titration. The primary endpoint was symptom change between titration initiation and completion. Secondary outcomes included titration characteristics, patient-reported satisfaction with symptom management, and adverse events.

**Results:**

All patients had cancer, and 33 patients (87%) were receiving baseline opioid therapy. Titration was performed mainly for pain (89%, *n* = 34). Median pain intensity decreased from 8 to 2 on the NRS (*p* < 0.001), corresponding to a median reduction of 5 points. Overall, 34 patients (90%) were classified as responders. Dyspnea improved in all 4 treated patients. Clinically relevant adverse events occurred in 4 patients (11%). At 24 h, 29 patients (76%) reported complete satisfaction with symptom management. Greater symptom reduction was associated with higher patient-reported satisfaction, whereas clinically relevant adverse events were associated with lower satisfaction.

**Conclusion:**

In this real-world cohort of specialist palliative care patients, rapid intravenous opioid titration was associated with substantial symptom improvement, high patient satisfaction, and a low rate of clinically relevant adverse events during titration and the subsequent 20-min observation period. These findings support the feasibility and immediate tolerability of this approach but do not establish longer-term safety.

**Supplementary Information:**

The online version contains supplementary material available at https://doi.org/10.1007/s00520-026-11245-1.

## Introduction

Acute pain and dyspnea are common and distressing symptom crises in palliative care, often requiring rapid and effective management [1–4]. Opioids are recommended by international guidelines, including ESMO, ASCO, and EAPC, for the management of severe pain and dyspnea [2, 3, 5, 6]. Rapid opioid titration, based on repeated intravenous doses with close reassessment, aims to achieve prompt symptom control while monitoring for adverse effects. Previous studies have suggested that rapid intravenous opioid titration is effective and generally well tolerated, but the available evidence remains limited and is largely derived from small single-center cohorts [7–10]. Furthermore, contemporary data describing how rapid opioid titration is implemented in routine specialist palliative care practice—including dosing strategies, tolerability, and patient-reported outcomes—remain scarce. At the Centre Hospitalier Universitaire Vaudois (CHUV) Palliative and Supportive Care Service, rapid opioid titration is routinely used for acute pain or dyspnea. Despite its frequent use, these practices have not yet been systematically evaluated. The aim of this study was to describe the real-world implementation of rapid intravenous opioid titration in a specialist palliative care service and to evaluate symptom improvement, tolerability, and patient-reported satisfaction in patients experiencing acute pain or dyspnea crises.

## Methods

### Study design and patient population

This retrospective, single-center observational study was conducted at the Palliative and Supportive Care Service of Lausanne University Hospital (CHUV), Switzerland. We included consecutive adult inpatients with palliative care needs who underwent rapid intravenous opioid titration for an acute severe pain or dyspnea crisis between April 1, 2024, and February 24, 2026, as part of routine clinical practice within the Palliative and Supportive Care Service.

Eligible patients were aged 18 years or older, conscious, and able to self-report symptom intensity at the time of titration. Acute symptom crises were defined as either a new onset of severe pain or dyspnea or a clinically significant exacerbation of a pre-existing symptom requiring rapid intravenous opioid titration, as determined by the treating physician. When multiple titration episodes occurred in the same patient, only the first episode was included in the analysis.

Patients were excluded if relevant clinical data were unavailable in the medical record or if they had refused the use of their health-related data for research purposes.

Clinical data were extracted retrospectively from the electronic medical record system, pseudonymized, and stored on a secure password-protected institutional server. The patient selection process is presented in Supplementary Fig. 1.

The study was approved by the local ethics committee (CER-VD, ID: 2026–00723, May 5th, 2026). For deceased patients without documented consent and without prior refusal, authorization for reuse of health-related data was granted by the ethics committee in accordance with Swiss regulations.

## Data collection

Baseline characteristics included age, sex, cancer diagnosis and stage, comorbidity burden, baseline opioid therapy, and indicators of renal and hepatic function. Renal function was assessed using serum creatinine, KDIGO criteria for acute kidney injury, and CKD-EPI estimated glomerular filtration rate (eGFR), including eGFR values over the three months preceding titration. Hepatic function was assessed using the Child–Pugh classification (Supplementary Tables 1–4). Comorbidity burden was assessed using the Charlson Comorbidity Index (CCI).

Time-related variables included T0 (time of first intravenous opioid bolus), TEnd (time of final clinical assessment marking completion of titration), assessment 20 min after TEnd, assessment 24 h after TEnd, and titration duration (time interval between T0 and TEnd, expressed in minutes).

Treatment-related variables included baseline opioid therapy (molecule, route of administration, and daily dose expressed as oral morphine equivalents [OME]) and rapid titration characteristics, including opioid used, initial dose in intravenous morphine equivalent (IME), dose modifications, cumulative dose and number of boluses. Opioid conversion ratios are reported in Supplementary Table 5 and were used solely for analytical standardization.

Pain intensity was assessed using the Numerical Rating Scale (NRS; 0–10), while dyspnea intensity was assessed using the Verbal Descriptor Scale (VDS; mild, moderate, severe). Symptom assessments were recorded at T0 and TEnd. Responders were defined as patients experiencing a reduction of at least 2 points on the NRS or an improvement of at least one category on the VDS.

Patient-reported satisfaction with symptom management was assessed 24 h after titration by direct questioning during routine clinical assessment and categorized as complete, partial, or absent satisfaction according to documentation in the medical record. Titration success was defined as completion of the titration procedure due to symptom relief, without interruption because of adverse events or insufficient efficacy.

Adverse events occurring during titration and within 20 min after completion were systematically assessed, including somnolence, nausea, vomiting, xerostomia, dizziness, respiratory depression, and confusion, and graded according to CTCAE version 5.0. Clinically relevant adverse events were defined as grade ≥ 2 events and/or events leading to premature discontinuation of titration.

### Intravenous titration procedure

Rapid intravenous opioid titration was individualized according to previous opioid exposure. In patients already receiving opioids, the titration opioid generally corresponded to the baseline opioid molecule administered intravenously. Initial bolus doses were derived from the patient's baseline opioid regimen. In opioid-naive patients, titration was generally initiated with low-dose intravenous morphine. Additional intravenous boluses could be administered every 2 min when required, with clinical reassessment after each administration. Titration was performed under close bedside supervision by the palliative care physician and nursing staff. Symptom intensity and treatment tolerability were evaluated throughout the procedure, and subsequent dose adjustments were individualized according to symptom response and adverse effects. Titration was continued until adequate symptom relief was achieved, clinically relevant adverse events occurred, or no meaningful symptom improvement was observed despite further dose escalation.

### Study objectives

The primary objective of this study was to assess short-term symptom outcomes following rapid intravenous opioid titration in palliative care patients experiencing acute severe pain and/or dyspnea crises.

The primary endpoint was the change in symptom intensity between T0 and TEnd, measured using the NRS for pain and the VDS for dyspnea.

Secondary objectives were to describe real-world titration practices, including opioid selection, cumulative opioid dose, number of boluses, and titration duration; to assess patient-reported satisfaction with symptom management 24 h after titration; and to evaluate treatment tolerability through the occurrence of clinically relevant adverse events.

### Statistics

Continuous variables were summarized using medians and interquartile ranges (IQR), and categorical variables as counts and percentages.

Changes in pain intensity between T0 and TEnd were analyzed using the Wilcoxon signed-rank test. Given the small number of patients treated for dyspnea, changes in VDS scores were described using category distributions.

Comparisons between independent groups (responders vs non-responders, patients with vs without clinically relevant adverse events, and satisfied vs non-satisfied patients) were performed using the Mann–Whitney U test for continuous variables and Fisher’s exact test for categorical variables.

Associations between clinically relevant adverse events and titration characteristics, including cumulative opioid dose and titration duration, were explored using the Mann–Whitney U test. Exploratory analyses also examined the relationship between baseline patient characteristics (age and Charlson Comorbidity Index) and the occurrence of clinically relevant adverse events.

Given the exploratory nature of the study and the limited sample size, all secondary and subgroup analyses should be considered hypothesis-generating.

All statistical tests were two-sided, and a p-value < 0.05 was considered statistically significant. Analyses were performed using JASP (version 0.96).

## Results

38 patients were included. Median age was 63 years (IQR 16), and 47% (*n* = 18) were female. At the time of data collection, 82% (*n* = 31) had died. Although not an inclusion criterion, all patients had an underlying malignancy, with 68% (*n* = 26) presenting with stage IV disease. The most frequent primary tumor sites were upper gastrointestinal, urogenital, and lung cancers (each 21%, *n* = 8). Median CCI was 8 (IQR 3).

Chronic kidney disease was present in 4 patients (11%), including 3 patients with CKD G3a, and 1 with CKD G3b. Three patients had acute kidney injury, including two with KDIGO stage 1 and one with KDIGO stage 3. One patient with stage 1 acute kidney injury had underlying chronic kidney disease and experienced confusion, while the other patient with stage 1 acute kidney injury and the patient with stage 3 acute kidney injury had no documented adverse events during the observed period. Liver failure was uncommon (*n* = 1).

Most patients (87%, *n* = 33) were receiving baseline opioid therapy, predominantly morphine (66%, *n* = 25), with a median oral morphine equivalent dose of 80 mg/day (IQR 90) (Table 1).

**Table 1:** Baseline characteristics

	Absolute frequency (Relative frequency)*
Age* (yo)	63 (16)
*Sex*	
*Female*	18 (47%)
*Male*	20 (53%)
*Vital Status at data collection*	
*Deceased*	31 (82%)
*Alive*	7 (18%)
*Titration Set up*	
*Palliative inpatient consultation*	22 (58%)
*Palliative care admission*	16 (42%)
*Cancer location*	
*Upper GI*	8 (21%)
*Urogenital*	8 (21%)
*Lung*	8 (21%)
*ENT*	7 (18%)
*Breast*	3 (9%)
*Lower GI*	2 (5%)
*Hematological*	2 (5%)
Cancer stage IV**	
*Yes*	26 (68%)
*No*	12 (32%)
*Charlson Comorbidity Index*	8 (3)
*Liver function impairment*	
* Child–Pugh C*	1(3%)
*Kidney function impairment*	
*Chronic Kidney Disease*	
*G3a*	3 (8%)
*G3b*	1 (3%)
*Acute Kidney Injury°*	
*KDIGO 1*	2 (5%)
*KDIGO 3*	1 (3%)
*Baseline Opioid*	
*Type*	
*Morphine*	25 (66%)
*Hydromorphone*	4 (11%)
*Fentanyl*	3 (8%)
*Methadone*	1 (3%)
*None*	5 (12%)
*Route*	
*PO*	18 (47%)
*IV*	11 (29%)
*SC*	3 (8%)
*TDM*	1 (3%)
*None*	5 (13%)
OME Dose (mg/d)*	80 (90)

*for continuous variables, results presented as median (IQR)

**According to TNM scoring system 8th or 9th

*°One patient with KDIGO stage 1 acute kidney injury had underlying chronic kidney disease *

*GI *gastro-intestinal*, ENT *ears nose throat*, PO *oer Os*, IV *intravenous*, SC *subcutaneous*, TDM *transdermal*, OME *oral daily morphine equivalent*, IQR *interquartile range

Rapid intravenous opioid titration was performed mainly for pain (89%, *n* = 34), while four patients (11%) underwent titration for dyspnea. Morphine was the most frequently used opioid (76%, *n* = 29).

The median initial dose was 5 mg IME (IQR 2), with a median of 3 boluses administered (IQR 2). Median cumulative opioid exposure was 12 mg IME (IQR 6), and median titration duration was 8 min (IQR 4) (Table 2).

**Table 2:** Titration characteristics

		Absolute frequency (Relative frequency) *	Absolute frequency (Relative frequency) *
*Indication for titration *			
	*Dyspnea*	4 (11%)	4 (11%)
	*Pain*	34 (89%)	34 (89%)
*Titration opioid (type)*			
	*Morphine*	29 (76%)	29 (76%)
	*Hydromorphone*	5 (13%)	5 (13%)
	*Fentanyl*	3 (8%)	3 (8%)
	*Methadone *	1 (3%)	1 (3%)
IME Initial dose* (mg)	5 (2)		
Titration bolus number*	3 (2)		
IME cumulative dose (mg)*	12 (6)		
Titration duration (minutes)*	8 (4)		
*Anti-emetic premedication*	17 (45%)		

* For continuous variables, results presented as median (IQR)

*IME *intravenous morphine equivalent

Among patients treated for pain, median NRS decreased from 8 (IQR 2) at baseline to 2 (IQR 3) at titration completion (p < 0.001), corresponding to a median reduction of 5 points (IQR 3).

Among the four patients treated for dyspnea, symptom intensity improved in all cases, with a shift from predominantly severe dyspnea at baseline to predominantly mild dyspnea following titration. Overall, 90% (*n* = 34) of patients met the predefined responder criteria (i.e. experiencing a reduction of at least 2 points on the NRS or an improvement of at least one category on the VDS). Titration was successfully completed in 82% (*n* = 31) of patients, the remaining 7 cases corresponding to 4 clinically relevant adverse events and 3 discontinuations due to insufficient symptom relief (Table 3).

**Table 3:** Clinical outcomes

	Absolute frequency (Relative frequency)*
*Pain (n=34)*	
Intensity at T0 (NRS)*	8 (2)
Intensity at TEnd (NRS)*	2 (3)
Change in NRS (T0–TEnd)*	5 (3)
*Dyspnea (n=4)*	
*Intensity at T0*	
* Severe*	3 (75%)
* Moderate*	1(25%)
*Intensity at TEnd*	
* Mild*	3 (75%)
* Moderate*	1(25%)
*Overall (n=38)*	
*Responder at TEnd *	34 (90%)
*Titration success at TEnd *	31 (82%)
*Complete Patient Satisfaction 24 hours after TEnd *	29 (76%)

*For continuous variables, results presented as median (IQR)

*NRS* numerical rating scale, *T0* time of first intravenous opioid bolus, *TEnd* time of titration’s completion

Adverse events were reported in 39% (*n* = 15) of patients and were predominantly grade 1. The most common events were somnolence (26%, *n* = 10) and dizziness (11%, *n* = 4). One patient experienced grade 3 somnolence, which resolved with observation alone. Clinically relevant adverse events occurred in 11% (*n* = 4) of patients, including 1 patient with grade 3 somnolence and 3 patients with grade 1 adverse events leading to titration discontinuation (Table 4, Supplementary Table 6).

**Table 4:** Adverse events characteristics

		*Absolute frequency (Relative frequency)*
Adverse event		
	No	23 (61%)
	Yes	15 (39%)
Type of adverse event		
	Somnolence	10 (26%)
	Dizziness	4 (11%)
	Confusion	1 (3%)
	Nausea	0 (0%)
	Vomiting	0 (0%)
	Xerostomia	0 (0%)
	Respiratory depression	0 (0%)
Grade^§^		
	Grade 1	14 (93%)
	Grade 3	1 (7%)
Clinically relevant AE°		
	No	34 (89%)
	Yes	4 (11%)

*Patients could experience more than one adverse event; therefore, individual adverse-event counts may exceed the number of patients with at least one adverse event*

*§Percentages for adverse event grades are calculated among patients experiencing an adverse event (n=15)*

*All grade 1 according to CTCAE 5.0, except one somnolence grade 3

*°AE leading to cessation of titration and/or ≥ Grade 2 according to CTCAE 5.0*

*AE *adverse event

At 24 h, 76% (*n* = 29) of patients reported complete satisfaction with symptom management.

Patients reporting complete satisfaction with symptom management experienced greater symptom improvement than those who were only partially satisfied or unsatisfied (median change in NRS 5 vs 2, *p* = 0.03). Conversely, clinically relevant adverse events were associated with lower satisfaction levels (*p* = 0.035) (Table 3).

No significant associations were observed between treatment response and titration characteristics or baseline patient characteristics.

## Discussion

In this retrospective study, rapid intravenous opioid titration was associated with substantial symptom relief in palliative care patients presenting with acute pain or dyspnea. Pain intensity decreased markedly, with a median reduction of 5 points on the NRS and a high proportion of responders (90%). Although only 4 patients underwent titration for dyspnea, all experienced symptom improvement, consistent with the established role of opioids in refractory breathlessness. Importantly, more than three quarters of patients reported complete satisfaction with symptom management 24 h after titration, suggesting that the observed symptom improvement translated into meaningful clinical benefit.

An important finding of our study is the rapidity with which symptom control was achieved. Patients required a median of only 3 intravenous boluses, corresponding to a median titration duration of 8 min. These findings suggest that rapid intravenous opioid titration can be readily performed at the bedside by specialist palliative care physicians and integrated into routine clinical practice. Because this study reflects daily practice rather than a protocolized research setting, it provides valuable real-world data regarding the feasibility of this approach.

Short-term tolerability was acceptable, with most adverse events being mild and clinically relevant adverse events occurring in only 4 patients (11%). One patient experienced grade 3 somnolence, which resolved with observation alone. Despite the high prevalence of baseline opioid use, the cumulative opioid dose administered during titration remained modest and was achieved with a limited number of boluses. Together with the low incidence of clinically relevant adverse events observed during titration and the subsequent 20-min period, these findings suggest acceptable immediate tolerability under close clinical supervision.

In addition, we found that greater symptom improvement was associated with higher patient-reported satisfaction with symptom management, whereas clinically relevant adverse events negatively affected patient perceptions, emphasizing the importance of balancing efficacy and tolerability.

Our findings are consistent with previous reports of rapid intravenous opioid titration in advanced cancer patients. Studies using either morphine or oxycodone have shown meaningful pain relief within minutes and a low incidence of significant toxicity, in agreement with the efficacy and immediate tolerability outcomes observed in our cohort [7, 9, 10].

Several limitations should be acknowledged. The retrospective design may introduce selection and information bias. The sample size was small, particularly for dyspnea, limiting statistical power and generalizability. In addition, the study was conducted in a single tertiary center, which may restrict external validity. Moreover, the absence of standardized titration protocols reflects real-world practice but may introduce variability. Because of the observational design, placebo effects cannot be excluded. Furthermore, adverse events were systematically assessed only during titration and for 20 min thereafter, and patient-reported satisfaction with symptom management was assessed at 24 h. Therefore, delayed opioid-related toxicity, including delirium occurring beyond this time frame, may have been missed. In addition, subsequent changes to patients’ baseline opioid regimens following titration were not systematically collected and may have influenced symptom control and patient satisfaction at 24 h. Consequently, patient-reported satisfaction with symptom management assessed at 24 h cannot be attributed exclusively to the rapid titration procedure. Further prospective studies are warranted to better characterize the efficacy and optimal use of rapid intravenous opioid titration. However, placebo-controlled studies in patients experiencing severe pain or dyspnea raise important ethical concerns and may prove difficult to conduct. Because all included patients had cancer, extrapolation to patients with non-malignant serious illnesses should be undertaken cautiously.

Despite these limitations, our findings provide real-world evidence supporting the association with symptom improvement, tolerability, and feasibility of rapid intravenous opioid titration for acute symptom crises in specialist palliative care.

## Conclusion

In this real-world cohort, rapid intravenous opioid titration was associated with substantial and rapid symptom improvement, high patient-reported satisfaction with symptom management, and a low rate of clinically relevant adverse events during titration and the subsequent 20-min observation period. These findings support the feasibility and immediate tolerability of this approach in specialist palliative care settings but do not establish longer-term safety.

## Supplementary Information

Below is the link to the electronic supplementary material.

ESM 1 (DOCX 48.2 KB)

## Data Availability

The data that support the findings of this study are not publicly available due to privacy and ethical restrictions related to patient confidentiality. De-identified data may be made available from the corresponding author upon reasonable request and subject to approval by the relevant ethics committee and institutional regulations.
