# Supplementary material for: Rapid intravenous opioid titration for acute symptom crises in palliative care: a real-world retrospective study
Source: Support Care Cancer. 2026 Sep 23;34(10):1010. doi: 10.1007/s00520-026-11245-1 (PMC13601182; doi:10.1007/s00520-026-11245-1)
Supplement: Supplementary file 1 [file 520_2026_11245_MOESM1_ESM.docx]

**SUPPLEMENTARY MATERIALS**

**Patient selection and inclusion**

*Figure S1. Flow chart diagram*

**Flow chart of patient inclusion**

Identification through Soarian database

Records screened

(n = 38)

GC documented

(n = 25)

**Identification**

**Screening**

**Included**

Included and analysed

(n =38)

No documented GC

(n = 13)

*Deceased ; no documented refusal ; data reuse authorized by CER-VD*

*GC: General Consent*

Flowchart of study population. A total of 38 patients were screened and included in the study. All 38 patients were analyzed. Among them, 25 had documented General Consent. Thirteen patients were deceased, had no documented refusal of health-related data reuse, and their inclusion and data reuse were authorized by the institutional ethics committee (CER-VD, ID: 2026-00723, May 5th, 2026).

**Kidney failure definition**

*Table S1. Acute kidney Injury Definition according to KDIGO 2012 Classification(1)*

| Stage | Serum creatinine criteria | Urine output criteria |
| --- | --- | --- |
| ****Stage 1**** | Increase ≥ 26.5 µmol/L within 48 h **or** 1.5–1.9 × baseline | < 0.5 mL/kg/h for 6–12 h |
| ****Stage 2**** | 2.0–2.9 × baseline | < 0.5 mL/kg/h for ≥ 12 h |
| ****Stage 3**** | 3.0 × baseline **or** ≥ 353.6 µmol/L **or** initiation of renal replacement therapy | < 0.3 mL/kg/h for ≥ 24 h **or** anuria ≥ 12 h |

Acute kidney failure was defined according to the Kidney Disease : Improving Global Outcomes (KDIGO) criteria for acute kidney injury (AKI), based on serum creatinine changes and urine output. In this study, classification was primarily based on serum creatinine due to data availability.

**Hepatic failure definition**

*Table S2. Child Pugh Score(2)*

| Parameter | 1 point | 2 points | 3 points |
| --- | --- | --- | --- |
| Total Bilirubin | < 34 µmol/L | 34–50 µmol/L | > 50 µmol/L |
| Serum albumin | > 35 g/L | 28–35 g/L | < 28 g/L |
| INR | < 1.7 | 1.7–2.3 | > 2.3 |
| Ascites | None | Mild | Severe/refractory |
| Hepatic encephalopathy | None | Grade I–II | Grade III–IV |

| Total Score | Class | Clinical Severity | 1-Year Survival |
| --- | --- | --- | --- |
| 5–6 points | Child-Pugh A | Compensated cirrhosis | **85–100%** |
| 7–9 points | Child-Pugh B | Moderately decompensated cirrhosis | **60–80%** |
| 10–15 points | Child-Pugh C | Severely decompensated cirrhosis | **35–45%** |

*INR: International Normalized Ratio*

*Table S3. Hepatic encephalopathy grade according to West Haven classification(2)*

| Grade | Clinical Features | Cognitive / Behavioral Changes |
| --- | --- | --- |
| Grade 0 | No detectable changes | Normal consciousness, normal psychometric tests |
| Grade I | Mild symptoms, often subtle | Trivial lack of awareness, shortened attention span, impaired addition or subtraction, sleep disturbances, mild confusion |
| Grade II | Moderate encephalopathy | Lethargy, disorientation to time, obvious personality changes, inappropriate behavior, asterixis present |
| Grade III | Severe encephalopathy | Marked confusion, disorientation to place, somnolence but arousable, incoherent speech, significant behavioral abnormalities |
| Grade IV | Coma | Unresponsive to pain, no meaningful verbal response |

Hepatic function was assessed using the Child–Pugh classification system. This score evaluates the severity of chronic liver disease based on five parameters: total bilirubin, serum albumin, International Normalized Ratio (INR), and the presence of ascites and hepatic encephalopathy. Patients were categorized into Child–Pugh classes A, B, or C, reflecting increasing severity of liver dysfunction.

**The Charlson Comorbidity Index**

*Table S4. The Charlson Comorbidity Index (3,4)*

| Condition | Details | Score |
| --- | --- | --- |
| Myocardial infarction | History of definite or probable myocardial infarction (EKG changes and/or enzyme changes) | 1 |
| Congestive heart failure | Exertional or paroxysmal nocturnal dyspnea and has responded to digitalis, diuretics, or afterload reducing agents | 1 |
| Peripheral vascular disease | Intermittent claudication or past bypass for chronic arterial insufficiency, history of gangrene or acute arterial insufficiency, or untreated thoracic or abdominal aneurysm (≥6 cm) | 1 |
| Cerebrovascular disease | Stroke or transient ischemic accident | 1 |
| Dementia |  | 1 |
| Chronic pulmonary disease |  | 1 |
| Connective tissue disease |  | 1 |
| Peptic ulcer disease |  | 1 |
| Liver disease | Mild: chronic hepatitis (or cirrhosis without portal hypertension) | 1 |
|  | Moderate: cirrhosis and portal hypertension but no variceal bleeding history | 2 |
|  | Severe: cirrhosis and portal hypertension with variceal bleeding history | 3 |
| Diabetes mellitus | Without complications | 1 |
|  | With end-organ damage | 2 |
| Hemiplegia or paraplegia |  | 2 |
| Moderate or severe renal disease | Severe = on dialysis, status post kidney transplant, uremia  Moderate = creatinine >0.27 mmol/L | 2 |
| Solid tumor | Without metastasis | 2 |
|  | Metastatic | 6 |
| Leukemia |  | 2 |
| Lymphoma |  | 2 |
| AIDS |  | 6 |
| Age | 50–59 years | 1 |
|  | 60–69 years | 2 |
|  | 70–79 years | 3 |
|  | ≥80 years | 4 |

*AIDS: Acquired Immunodeficiency Syndrome*

Comorbid burden was assessed using the Charlson Comorbidity Index (CCI). This validated score predicts mortality risk by weighting a range of predefined comorbid conditions, including cardiovascular, respiratory, renal, and oncological diseases. The final score is obtained by summing weighted comorbidities, with higher values indicating greater overall disease burden and worse prognosis.

**Opioid conversion table**

*Table S5. Opioid conversion table(5)*

| Opioid | Ratio conversion |
| --- | --- |
| Oral Morphine  to parenteral morphine  (I.V./S.C.) | 3 :1 |
| Morphine to hydromorphone | 5 :1 |
| Oral Morphine  to oral oxycodone | 2 : 1 |
| Oral morphine to  Oral methadone | < 100 mg morphine  7 :1  100 – 199 mg morphine  9 : 1  >200 mg morphine  12 : 1 |
| Oral methadone to  parenteral methadone | 1 : 1 |
| Transdermic fentanyl  To oral morphine | 1 :100 |

*I.V. : Intravenous, S.C: Subcutaneous*

Opioid doses were standardized using an equianalgesic conversion table to allow comparison across different opioid molecules. All administered opioids were converted into morphine milligram equivalents using validated equianalgesic ratios. This approach ensures comparability of opioid exposure between patients and across treatment regimens.

**Clinically relevant adverse events**

*Table S6. Patients with and without clinically relevant adverse events*

| Variable | No clinically relevant AE (n=34)  *Median (IQR)* | Clinically relevant AE (n=4)  *Median (IQR)* |
| --- | --- | --- |
| Age | 63.5 (15) | 60 (13) |
| Sex (Female/male) | 16 (47.1%) / 18 (52.9%) | 2 (50%) /2 (50%) |
| CCI | 8 (3) | 8 (1.3) |
| Baseline OME (mg) | 80 (89) | 60 (113) |
| Initial IME dose (mg) | 5 (2.4) | 5.5 (5.4) |
| Cumulative IME dose (mg) | 10.5 (5.8) | 13.3 (4.1) |
| Number of boluses | 3 (2) | 3 (2.3) |
| Titration duration (min) | 8 (2.8) | 9 (4.8) |

*Categorical variables are presented as absolute frequency (relative frequency). Continuous variables are presented as median (IQR).*

*AE : Adverse Event, CCI : Charlson Comorbidity index, OME : Oral Morphine Equivalent, IME : Intravenous Morphine Equivalent*

Supplementary Table S6 compares baseline and titration characteristics of patients with and without clinically relevant adverse events. Given the small number of patients experiencing clinically relevant adverse events (n=4), results are presented descriptively and should be considered exploratory.

**Bibliography**

1.	Khwaja A. KDIGO clinical practice guidelines for acute kidney injury. Nephron Clin Pract. 2012;120(4):c179-84. doi: 10.1159/000339789. Epub 2012 Aug 7. PMID: 22890468.

2.	Hepatic Encephalopathy in Chronic Liver Disease: 2014 Practice Guideline by the European Association for the Study of the Liver and the American Association for the Study of Liver Diseases. Journal of Hepatology. sept 2014;61(3):642‑59. doi:10.1016/j.jhep.2014.05.042

3.	Quan H, Li B, Couris CM, Fushimi K, Graham P, Hider P, et al. Updating and Validating the Charlson Comorbidity Index and Score for Risk Adjustment in Hospital Discharge Abstracts Using Data From 6 Countries. American Journal of Epidemiology. 15 mars 2011;173(6):676‑82. doi:10.1093/aje/kwq433

4.	Extermann M. Measuring comorbidity in older cancer patients. European Journal of Cancer. mars 2000;36(4):453‑71. doi:10.1016/S0959-8049(99)00319-6

5.	Davis MP, Davies A, McPherson ML, Reddy AS, Paice JA, Roeland EJ, et al. Opioid conversion in adults with cancer: MASCC-ASCO-AAHPM-HPNA-NICSO guideline. Support Care Cancer. mars 2025;33(3):243. doi:10.1007/s00520-025-09286-z
